# Editorial

**Published:** 2012-10-16

**Authors:** Shibal Bhartiya, Tarek Shaarawy, Tanuj Dada

Dear Friends,

Warm Greetings

The editorial team at Journal of Current Glaucoma Practice (JOCGP), the official Journal of International Society of Glaucoma Surgery, hopes to chronicle new developments in the field of glaucoma for you, ensuring a critical evaluation of existing and new techniques for better patient care. We are indeed grateful to the dynamic and illustrious editorial board for agreeing to join us in this endeavor.

In this issue, Coote et al review the techniques of bleb revision for failing blebs. Stirbu et al review various materials and modalities for drainage tube repair.

The role of selective laser trabeculoplasty and prostaglandin analogs in the current glaucoma treatment paradigm is discussed by Clement, in the perspectives section. Louati et al draw attention to the implications of long-term BAK use in patients of a chronic disease like glaucoma.

Nuyen et al introduce the technique of imaging the lamina cribrosa using the swept-source optical coherence tomography in the diagnostics section. Maheshwari et al review the methods and implications of tonometry in glaucoma while assessment of diurnal fluctuations in intraocular pressure is dealt with, by Bhartiya et al.

Ichhpujani et al discuss the extremely important assessment of knowledge, attitudes and self-care practices associated with glaucoma in hospital personnel.

We hope you enjoy reading this issue of the JOCGP and will always welcome any comments, criticisms or suggestions that you may have.

Looking forward to seeing you all at Glasgow in September.

Best wishes

**Shibal Bhartiya**

**Tarek Shaarawy**

**Tanuj Dada**

